# Lyophilized Kit for the Preparation of the PET Perfusion Agent [^68^Ga]-MAA

**DOI:** 10.1155/2014/269365

**Published:** 2014-03-31

**Authors:** Alejandro Amor-Coarasa, Andrew Milera, Denny Carvajal, Seza Gulec, Anthony J. McGoron

**Affiliations:** ^1^Biomedical Engineering Department, Florida International University, 10555 West Flagler Street, EC 2614, Miami, FL 33174, USA; ^2^Herbert Wertheim College of Medicine, Florida International University, 1240 SW 108 Avenue, University Park, Miami, FL 33174, USA; ^3^Mount Sinai Medical Center, 4300 Alton Road, Miami Beach, FL 33140, USA

## Abstract

Rapid developments in the field of medical imaging have opened new avenues for the use of positron emitting labeled microparticles. The radioisotope used in our research was ^68^Ga, which is easy to obtain from a generator and has good nuclear properties for PET imaging. *Methods*. Commercially available macroaggregated albumin (MAA) microparticles were suspended in sterile saline, centrifuged to remove the free albumin and stannous chloride, relyophilized, and stored for later labeling with ^68^Ga. Labeling was performed at different temperatures and times. ^68^Ga purification settings were also tested and optimized. Labeling yield and purity of relyophilized MAA microparticles were compared with those that were not relyophilized. *Results*. MAA particles kept their original size distribution after relyophilization. Labeling yield was 98% at 75°C when a ^68^Ga purification system was used, compared to 80% with unpurified ^68^Ga. Radiochemical purity was over 97% up to 4 hours after the labeling. The relyophilized MAA and labeling method eliminate the need for centrifugation purification of the final product and simplify the labeling process. Animal experiments demonstrated the high *in vivo* stability of the obtained PET agent with more than 95% of the activity remaining in the lungs after 4 hours.

## 1. Introduction

Starting in 1964, several efforts have been made to find an agent for perfusion and embolization [1, 2]. A lyophilized kit for the preparation of ^99m^Tc-MAA was created in 1974 for Single Photon Emission Tomography (SPECT) imaging. With the arrival of Positron Emission Tomography (PET) the formulation of an analogue drug with a positron emitter was needed. Among the available PET isotopes ^68^Ga is easily obtained from its parent nuclide ^68^Ge by chromatographic column separation with different inorganic exchangers. The long lived parent allows the construction of a generator that can last up to two years [3] compared to a ^99m^Tc/^99^Mo generator which lasts only for 1-2 weeks. MAA was first successfully labeled with ^68^Ga in 1989 [4] but never used, probably due to unreliability of the existing ^68^Ge/^68^Ga generators and low availability of PET imaging cameras. Revived interest has been shown recently [5, 6], and the first PET lung perfusion studies in humans have been performed [7, 8].

Selective internal radiation treatment (SIRT), a technique used to treat metastatic liver cancer, could also benefit from a PET perfusion tracer. During the planning stage, a ^99m^Tc-MAA perfusion scan is performed to assess the allocation in lung and gastrointestinal tract. It is also used to calculate tumor to normal liver allocation ratio [9]. The distribution acts as a predictor of the treatment safety and effectiveness. A PET perfusion agent (e.g., ^68^Ga-MAA) could provide valuable, quantifiable information to calculate precise doses, which could potentially improve the treatment outcome. Initial work with ^68^Ga-MAA for SIRT planning has already been performed [10].

All reported ^68^Ga labeling of MAA has been done using a commercial MAA kit for ^99m^Tc (mainly Pulmolite kit, no longer on the market). All kits contain approximately 100 *μ*g stannous chloride and free albumin; hence the particles need to be washed with saline before ^68^Ga labeling. The maximum reported labeling yield using the Pulmolite kit is around 80% [5]. Elimination of the free ^68^Ga via centrifugation is therefore necessary. The purification process is also required to eliminate traces of the long half-life ^68^Ge that are eluted from the generator (required only if ^68^Ge ≥ 0.001% of the ^68^Ge/^68^Ga generator activity or 0.5 *μ*Ci for a 50 mCi generator). A new ^68^Ga specific MAA lyophilized kit is needed for labeling. Further, it needs to be combined with a prepurification system that assures prior elimination of ^68^Ge traces and provides pure, preconcentrated ^68^Ga for labeling.

## 2. Materials and Methods

### 2.1. MAA Lyophilized Kit Preparation

Macroaggregated albumin (MAA) was obtained from Triad Isotopes (DraxImage kit). The content was reconstituted with 0.9% saline solution, separated into two 15 mL centrifuge tubes (to match the original 4 · 10^6^ number of particles present in the Pulmolite kit for comparison purposes), centrifuged (Eppendorf, Germany), and the supernatant discarded (“washed MAA”) [4]. The particles were then relyophilized overnight and stored for labeling (“relyophilized MAA”). Size and morphology analysis was performed on reconstituted MAA using an optical microscope (Micromaster, Fisher-Sci, USA) and a hemacytometer (Reichert, USA) before and after relyophilization.

### 2.2. ^68^Ge/^68^Ga Radioisotopic Generator

The ^68^Ge/^68^Ga generator used was the 50 mCi IGG-100 (Eckert & Ziegler, Germany), based on the TiO_2_ resin technology, eluted with 5 mL of 0.1 M ultrapure HCl (Sigma-Aldrich, USA) solution.

### 2.3. Purification System

A combination of chromatographic exchange resins was used [11]. Two Luer-fitting column beds were prepared. First, 40 ± 10 mg of AG-50Wx8 cation exchange (Eichrom, USA) column is connected to a three-way stopcock. Next, 15 ± 5 mg of UTEVA anion exchange (Eichrom, USA) resin in a column is positioned. Finally, another three-way stopcock is located at the end. The system was designed to be used in four simple steps: elution (from the generator, 5 mL of 0.1 M HCl); cleaning (1 mL of 0.1 M HCl); purification (1 mL of 5 M HCl); extraction (1 mL of Millipore Water) (Figure 2).

### 2.4. MAA Labeling

Both, washed MAA and relyophilized MAA, were labeled (using the original Green's method [4, 5]) with either purified or unpurified ^68^Ga solution. The unpurified ^68^Ga solution was obtained directly from the generator (5 mL, pH = 1, A *≈* 10 mCi). The purified ^68^Ga elution (1 mL, pH = 0.6, A *≈* 10 mCi) was obtained from the purification system. Both were buffered using 0.3 mL of 3 N ultrapure sodium acetate (Sigma-Aldrich, USA). The solution was added to the 15 mL centrifuge tube containing the MAA. Labeling was performed using a thermomixer with a heating block for 15 mL centrifuge tubes and stirring at 750 rpm (Eppendorf, Germany). Labeling temperature was 25 (room temperature), 50, 75, and 95 degrees Celsius. Labeling time was set at 15 minutes based on previous reports of MAA-^68^Ga labeling kinetics [4, 5]. Particles were separated from the supernatant by centrifugation. The particles and supernatant were measured separately using an Atomlab 100 dose calibrator (Biodex, USA). Final particles were resuspended in 5 mL saline solution with a vortex mixer (Fisher-Sci, USA).

### 2.5. Animal Experiments

Sprague Dawley rats (200–225 grams, 2 per time point) were obtained from Harlan Laboratories (Harlan, USA). Animals were weighed before the procedure and anesthetized using an Ohmeda Isotec 3 isoflurane vaporizer (GE Healthcare, USA). Isoflurane levels were kept ≤3% at all times. Once completely anesthetized, animals were restrained in the supine position and a torso X-Ray was obtained (Belmont Acuray 071A, USA). Later, 100 *μ*L of the labeled MAA (8,000–10,000 particles) with an activity ranging from 50 to 100 *μ*Ci (1.85–3.7 MBq) was injected through the lateral tail vein. Animals were euthanized at 2 or 4 hours. For either time points their lungs, liver, spleen, heart, kidneys, ribs, and 0.2 mL of blood and urine were collected, weighed, and activity measured using a Cobra 5000 well counter (Packard, USA). Uncollimated autoradiography images (in the unaltered supine position the X-Ray was obtained) were also taken at 1, 2, 3, and 4 hours (Packard Phosphorimager, Perkin Elmer, USA). Free ^68^Ga was injected as a control. Additionally, imaging and organ collection were also performed with ^99m^Tc-MAA and reduced Na ^99m^TcO_4_ (sodium pertechnetate) for comparison purposes ^99m^Tc-MAA and Na ^99m^TcO_4_ were purchased from a local pharmacy (Triad Isotopes, USA). Approximately 5 mCi of sodium pertechnetate in 5 mL of saline (pH *≈* 5.5) was reduced with 100 *μ*g of stannous chloride (Sigma-Aldrich, USA) before injection. The obtained X-Rays and the autoradiography images were superimposed to provide anatomical and functional data.

## 3. Results and Discussion

### 3.1. MAA Relyophilization

**Figure 1:**
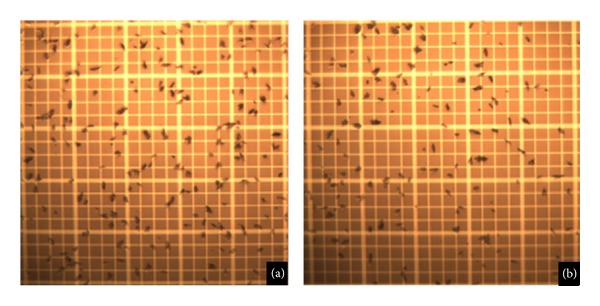
MAA microscope images, (a) from original unmodified MAA kit and (b) from relyophilized MAA.

The elimination of the excess free albumin is a necessary step prior to successful labeling with ^68^Ga (“washed MAA”) [4, 5]. Resuspension of the particles was fast using a vortex mixer; manual shaking of the vial was also efficient. Relyophilization of the MAA did not change either the particle's size distribution or morphology (Figure 1).

### 3.2. Purification System

**Figure 2:**
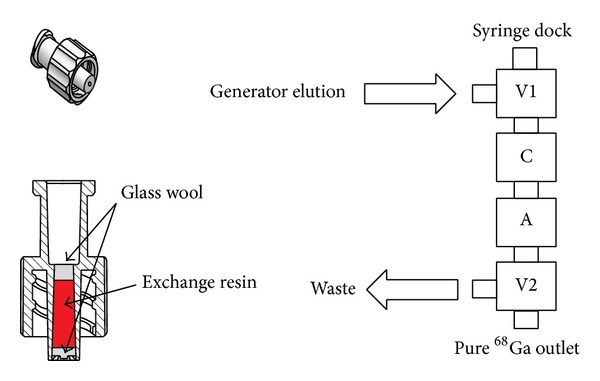
Purification System Setup.

The assembly of the purification system is simple and easy to use (Figure 2).

**Figure 3:**
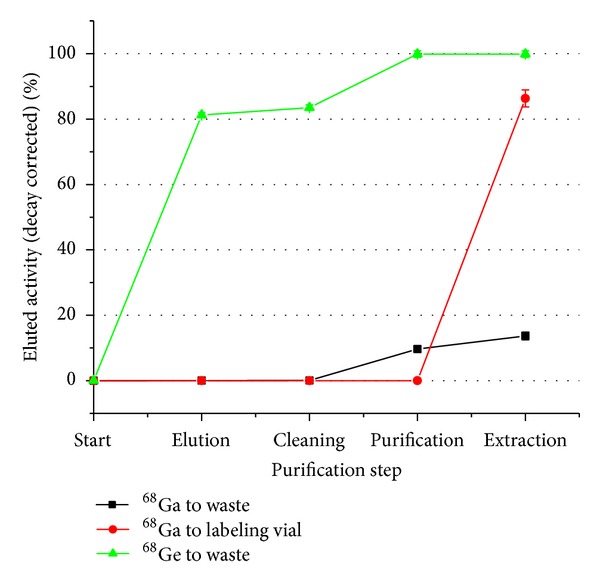
^68^Ga recovery and ^68^Ge elimination during the purification process.

The optimized and simple four-step purification process is as follows. (1)* Elution* of the generator using a 0.1 M HCl solution. During this step the gallium is trapped with most of the metal impurities in the cation exchanger. Most of the ^68^Ge contamination is removed in this step. (2)* Cleaning* is performed with one extra mL of 0.1 M HCl through the syringe dock to remove the excess solution coming from the generator. One mL of air is then vented to the system. (3)* Purification* is performed with slow elution using 1 mL of 5 M HCl. The Ga^3+^ forms a GaCl_4_^−^ complex so it is released from the cation exchanger and absorbed onto the anion exchanger. One mL of air is also used to vent the system. All the metal impurities are eliminated in this step (especially Fe, Ti, and Zn). (4)* Extraction* is done using 1 mL of Millipore water in which the gallium complex is destroyed and released from the anion exchanger into the labeling vial. One mL of air is pushed through the system to remove most of the ^68^Ga (Figure 3). The purification system retrieves approximately 85% of the eluted gallium activity after 10 minutes of processing. It provides pure, preconcentrated ^68^Ga in slightly acidic solution that is buffered by adding 0.3 mL of 3 N ultrapure sodium acetate [11]. All the ^68^Ge is eliminated in the process; consequently radio-nuclide impurities are eliminated from the labeling process.

The purification system can be reused for 100+ times without altering its performance. However to maintain sterility it is recommended to be used once and disposed.

### 3.3. MAA Labeling

**Figure 4:**
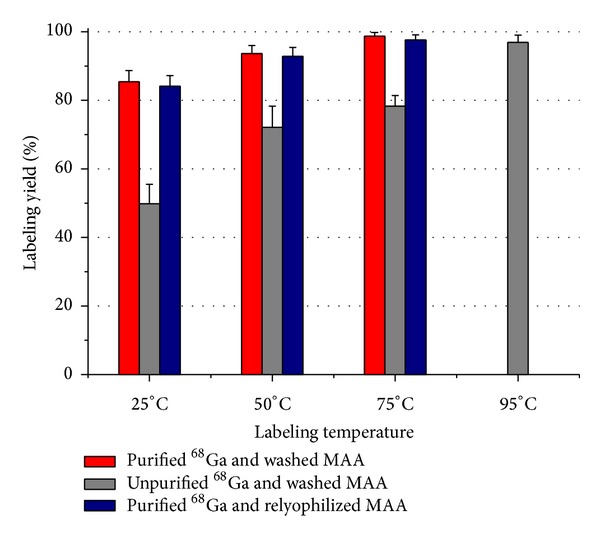
^68^Ga-MAA labeling yield results.

Labeling yield of MAA with unpurified ^68^Ga was 78.3 ± 3.1% after 15 minutes at 75°C, similar to that reported by other investigators [4]. A labeling yield of 72.1 ± 6.2% was obtained at 50°C (Figure 4). Better labeling yield (96.9 ± 2.1%) was obtained at 95°C; however the particle quality control showed important changes. Smaller particles were detected and in higher concentration, apparently due to the rupture of bigger macroaggregates. The labeling yield at room temperature was 50 ± 4%. Radiochemical purity tests were conducted for all the products showing more than 97% “*in vitro*” stability in all cases after 4 hours.

The introduction of the ^68^Ga purification system improved the labeling yield significantly (*P* < 0.003). An 84.1 ± 3.1% labeling yield was obtained at room temperature (25°C). This yield is higher than the maximum yield obtained at 75°C with unpurified ^68^Ga. However, if room temperature labeling is performed, postlabeling purification is still needed to assure a final radiochemical purity >90%. In the particular case of MAA, labeling at up to 75°C has been proven to not damage the particles. Nevertheless, synthesis near room temperature or elimination of the heating step all together is obviously desirable. Good labeling yield of 92.8 ± 2.6% was obtained at 50°C. When labeling at this temperature, purification is still recommended since nearly 10% of free ^68^Ga will be present in the final product.

The labeling of MAA with purified ^68^Ga yielded the best results at 75°C (Figure 4). A labeling yield of 97.6 ± 1.5% was obtained after 15 minutes of reaction. Particle distribution and morphology remained well within specifications and a >95% radiochemical purity was obtained. Labeling at this temperature eliminates the need for a purification step, rendering the final product ready for injection immediately. Experiments with purified ^68^Ga-MAA at 95°C were not performed because of the previously observed particle change at that temperature.

Relyophilized MAA labeling with purified ^68^Ga showed no significant difference from the results obtained with the washed MAA (*P* > 0.8). The elimination of the free albumin and the stannous chloride from the original formulation followed by relyophilization of the MAA does not compromise either resuspension or morphology (and size distribution) of the particles. The relyophilized MAA (or MAA prepared without SnCl_2_ and free albumin), in combination with the ^68^Ga purification system, allows for the preparation of a single-use lyophilized kit for the preparation of ^68^Ga-MAA. This kit can be used for Positron Emission Tomography in lung perfusion studies, selective internal radiation treatment (for liver cancer) planning, and other applications requiring perfusion imaging.

Since terminal studies were conducted, sterility of the final product was not a concern. However sterility of the final product is very important to have a successful kit. To maintain ^68^Ga-MAA sterility, ^68^Ga needs to be filtered through a 0.22 *μ*m filter before labeling. In the same way, the MAA kit must be produced albumin and SnCl_2_ free for ^68^Ga labeling and its sterility must be guaranteed. Final product sterility cannot be assured by filtration (since MAA particles are in the *μ*m range); therefore a parametric release is needed. All labeling procedures for human use must be conducted in a clean room.

### 3.4. Animal Experiments

More than 90% of the injected ^68^Ga-MAA activity was detected in the lungs after tail vein injection (seen in the image taken after 10 minutes, not shown) and until at least 4 hours after injection (Table 1). Less than 2% of ID/o activity was measured in any organ other than lungs after 2 and 4 hours. Most of the free ^68^Ga (>60%) remains in the blood after 4 hours (presumably as ^68^Ga-native transferrin complex). The remaining activity was extracted by the kidneys to the bladder (13%) or absorbed by the liver (15%). The* in vivo* radiochemical purity of ^68^Ga-MAA was greater than 97% during the studied period.

The behavior and “*in vivo*” radiochemical purity of ^99m^Tc MAA was different than that of ^68^Ga-MAA. ^99m^Tc was slowly released from the MAA and extracted by the kidneys into the urine (7.6 ± 1.3 after 2 hours and 12.3 ± 1.2 after 4 hours). Only 86.6 ± 0.7% of the decay corrected activity was found in the lungs after 2 hours, decreasing to 79.2 ± 1.5% at 4 hours.

**Figure 5:**
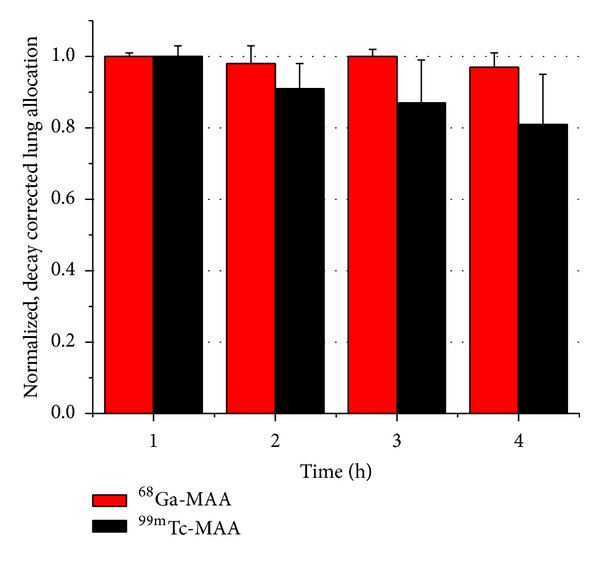
Normalized and radio-decay-corrected lung allocation for ^68^Ga-MAA and ^99m^Tc-MAA at 1, 2, 3, and 4 hours (*n* = 2 per time point).

**Figure 6:**
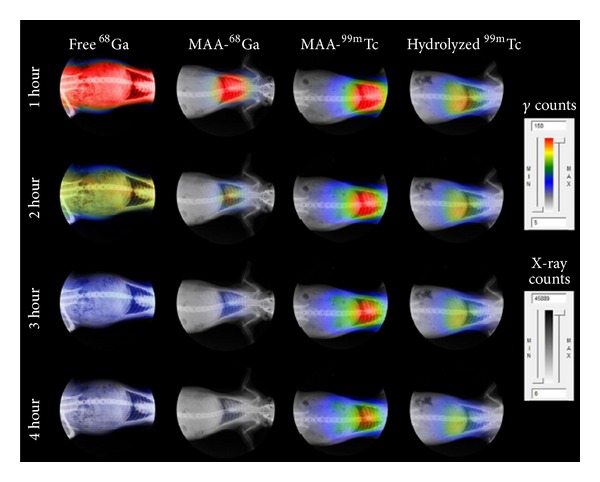
Non decay-corrected, uncollimated full body autoradiography for free ^68^Ga (*T*_1/2_ = 68 min), ^68^Ga-MAA, ^99m^Tc-MAA, and hydrolyzed ^99m^Tc (*T*_1/2_ = 6.02 h) at 1, 2, 3, and 4 hours. Labeling of ^68^Ga-MAA was performed with relyophilized MAA and purified ^68^Ga at 75°C for 15 minutes. The autoradiograph is superimposed on an X-ray image of the same animal in the unaltered supine position.

Hydrolyzed ^99m^Tc (reduced with SnCl_2_) allocates mainly in the lungs and liver. The % ID/o did not change over the study period. ^68^Ga-MAA exhibited better “*in vivo*” stability than ^99m^Tc-MAA (Table 1 and Figure 5). The autoradiography images clearly showed the preferential allocation of ^68^Ga-MAA in the lungs over the period studied (Figure 6). The drug-product* in-vivo* half-life was determined using regions of interest in the autoradiography images. A square cell of 40 × 40 mm was used to count the activity in the lung region for each time point (Figure 6). For ^99m^Tc-MAA biological half-live was found to be *T*_1/2_ = 11.4 ± 1.7 hours. This is consistent with the previously reported value of 11.5 ± 4 hour biological half-life [12]. The biological half-life of ^99m^Tc-MAA is not to be confused with the MAA biological half-life. These are equal only if 100% “*in vivo*” radiochemical purity of ^99m^Tc-MAA is assumed. The assumption was reinforced by the fact that injected hydrolyzed  ^99m^Tc behaves differently than that released from the MAA (Table 1 and Figure 6). The only feasible explanation is that injected hydrolyzed ^99m^Tc forms nanocolloids with the SnCl_2_, being absorbed by the liver and lungs, while ^99m^Tc released from the MAA is quickly absorbed by the kidneys. Furthermore, it is very unlikely that some form of degraded ^99m^Tc-albumin will be absorbed by the kidneys for excretion rather than be degraded in the liver. For over 40 years MAA half-life was considered to be in the 6–12-hour range [1, 12]. However if MAA half-life happened to be so short, degradation would have been observed in the ^68^Ga-MAA experiments. The stronger ^68^Ga-MAA binding, with superior “*in vivo*” stability, proves that MAA half-life is much longer than previously assumed [9].

The assumption of a shorter MAA half-life (MAA biological decomposition) has little or no implication in lung perfusion studies or probe guided surgery. However questionable implication is present when using the radiolabeled MAA in planning for liver cancer SIRT. The radio-microsphere technique is based on several planning steps. One of them is a particle distribution assessment using ^99m^Tc-MAA, mainly to determine lung and gastrointestinal (GI) allocation after hepatic artery injection. If only liver allocation is found (less than 20% lung allocation and no GI allocation), then the radio-microsphere treatment is administered after 48 hours since the MAA is assumed to have been cleared from the vessels (assuming a MAA half-life of 6–12 hours). Despite this wrong assumption, the treatment is successful. Therefore, it must be concluded that the effectiveness of the treatment does not require the complete decay of the treatment planning microparticles. Because they are injected in small numbers, enough arterioles seem to still be available for the allocation of the therapy particles. However, whether or not the treatment could benefit from the use of fast degrading planning particles (faster than MAA) remains an open question. Nevertheless, a precise determination of MAA half-life is needed and can probably be measured by combining the strong gallium binding with a longer radioactive half-life (e.g., ^67^Ga-MAA).

The relyophilization of washed MAA was a first approach to show the feasibility of a lyophilized kit specifically for ^68^Ga-MAA. In a production facility the pharmaceutical development would need to be different from that of the ^99m^Tc-MAA kit. Free albumin and SnCl_2_ would not need to be added to the final product. The high labeling yield obtained during the preparation of ^68^Ga-MAA eliminates the need for final centrifugation for purification. What seems to be the apparent elimination of a single step has major implications. In these conditions the purification/labeling scheme can be easily automated using one of the available modular labs for PET synthesis (e.g., Modular-Lab PharmTracer, Eckert and Ziegler, Germany), or it could easily be accomplished in a nuclear medicine hot-lab in a hospital.

## 4. Conclusions

A Gallium specific lyophilized kit for ^68^Ga-MAA production was created. The kit is comprised of a vial containing MAA (relyophilized DraxImage kit), a ^68^Ga purification system, and working solutions in the following syringes: 5 mL of 0.1 M HCl (elution), 1 mL 0.1 of M HCl (cleaning), 1 mL of 5 M HCl (purification), 1 mL of Millipore water (extraction), and 0.3 mL of 3 N NaAc solution (buffer). The purification system can also be used for labeling of most ^68^Ga imaging agents (e.g., DOTANOC and DOTATOC) with a small variation in the buffer amount. Labeling of MAA at 75°C for 15 minutes is recommended for labeling yields higher than 95% with no further purification necessary. Room temperature labeling is possible for producing 80% labeling yield but postlabeling purification is needed. Lung perfusion PET images are recommended to be acquired within the first hour after injection because of the short half-life of the ^68^Ga. The “*in vivo*” stability of the obtained ^68^Ga-MAA drug product is superior to that of ^99m^Tc-MAA. Use of ^68^Ga-MAA in SIRT planning is also possible, likely to benefit from superior imaging/quantification and more accurate dosimetric calculations.

## Figures and Tables

**Table 1:** Decay corrected organ biodistribution (DC-ID/o) of ^68^Ga-MAA, free ^68^Ga, ^99m^Tc-MAA, and hydrolyzed ^99m^Tc.

Organ	Free ^68^Ga		^ 68^Ga-MAA		^ 99m^Tc-MAA		Hydrolyzed ^99m^Tc	
	2 hours	4 hours	2 hours	4 hours	2 hours	4 hours	2 hours	4 hours
Spleen	0.6 ± 0.5	1.6 ± 0.1	0.1 ± 0.0	0.1 ± 0.0	0.1 ± 0.0	0.1 ± 0.0	7.2 ± 1.1	11.8 ± 0.7
**Blood**	**84.9 ± 4.5**	**63.1 ± 3.9**	**0.8 ± 0.8**	**0.8 ± 0.0**	**0.9 ± 0.2**	**0.9 ± 0.0**	**1.3 ± 1.2**	**0.4 ± 0.0**
Rib	0.5 ± 0.4	1.3 ± 0.4	0.0 ± 0.0	0.0 ± 0.0	0.0 ± 0.0	0.0 ± 0.0	0.0 ± 0.0	0.0 ± 0.0
Urine	6.8 ± 2.9	14.0 ± 1.7	0.1 ± 0.0	0.1 ± 0.0	7.6 ± 1.3	12.3 ± 1.2	6.1 ± 4.0	0.6 ± 0.2
Right kidney	0.5 ± 0.5	1.6 ± 0.4	0.0 ± 0.0	0.0 ± 0.0	2.1 ± 0.1	3.3 ± 0.1	0.6 ± 0.1	0.4 ± 0.0
Left kidney	0.5 ± 0.5	1.4 ± 0.2	0.0 ± 0.0	0.0 ± 0.0	2.2 ± 0.2	3.4 ± 0.2	0.6 ± 0.1	0.4 ± 0.0
Heart	0.7 ± 0.7	1.5 ± 0.2	0.0 ± 0.0	0.0 ± 0.0	0.0 ± 0.0	0.1 ± 0.0	0.2 ± 0.0	0.2 ± 0.0
**Lungs**	**3.1 ± 2.9**	**8.4 ± 0.3**	**98.6 ± 0.7**	**98.6 ± 0.1**	**86.6 ± 0.7**	**79.2 ± 1.5**	**39.5 ± 1.5**	**45.2 ± 10.9**
Liver	2.3 ± 1.9	7.2 ± 1.2	0.4 ± 0.2	0.4 ± 0.1	0.5 ± 0.0	0.9 ± 0.0	44.6 ± 2.8	40.9 ± 11.7

## Abbreviations

MAA: Macroaggregated albumin
PET: Positron Emission Tomography
SIRT: Selective Internal Radiation Treatment
SPECT: Single Photon Emission Tomography.

## References

[1] Furth ED, Okinaka AJ, Focht EF, Becker DV (1965). The distribution, metabolic fate and radiation dosimetry of ^131^I labeled macroaggregated albumin. *Journal of Nuclear Medicine*.

[2] Huberty JP (1971). 99mTc sulfur colloid absorbed on ferric hydroxide macroaggregates for lung perfusion imaging. *The International Journal Of Applied Radiation And Isotopes*.

[3] Schubiger PA, Lehmann L, Friebe M PET chemistry: the driving force in molecular imaging.

[4] Even GA, Green MA (1989). Gallium-68-labeled macroaggregated human serum albumin,68Ga-MAA. *International Journal of Radiation Applications and Instrumentation.*.

[5] Mathias CJ, Green MA (2008). A convenient route to [68Ga]Ga-MAA for use as a particulate PET perfusion tracer. *Applied Radiation and Isotopes*.

[6] Maus S, Buchholz H, Ament S, Brochhausen C, Bausbacher N, Schreckenberger M (2011). Labelling of commercially available human serum albumin kits with68Ga as surrogates for99mTc-MAA microspheres. *Applied Radiation and Isotopes*.

[7] Hofman MS, Beauregard J, Barber TW, Neels OC, Eu P, Hicks RJ (2011). 68Ga PET/CT ventilation-perfusion imaging for pulmonary embolism: a pilot study with comparison to conventional scintigraphy. *Journal of Nuclear Medicine*.

[8] Ament SJ, Maus S, Reber H (2013). PET lung ventilation/perfusion imaging using (68)Ga aerosol (Galligas) and (68)Ga-labeled macroaggregated albumin. *Recent Results in Cancer Research*.

[9] Gulec S, Mesoloras G, Dezarn W, McNeillie P, Kennedy A (2007). Biologic determinants of absorbed dose estimates in Y-90 microsphere treatment of hepatic malignancies: significance of tumor perfusion measured by Tc-99m MAA imaging. *Journal of Nuclear Medicine*.

[10] Gartenschlaeger M, Maus S, Buchholz H, Reber H, Pitton N, Schreckenberger M (2011). Investigation for extrahepatic shunt before SIRT by PET/CT with68Ga-MAA. *Nuklearmedizin*.

[11] Amor-Coarasa A, Gulec S, McGoron AJ (2012). Inexpensive and cGMP capable Ga-68 purification system. *Journal of Nuclear Medicine*.

[12] Chandra R, Shamoun J, Braunstein P, DuHov OL (1973). Clinical evaluation of an instant kit for preparation of (99m)Tc MAA for lung scanning. *Journal of Nuclear Medicine*.

